# The Impact of *Pseudomonas aeruginosa* Infection in Adult Cystic Fibrosis Patients—A Single Polish Centre Study

**DOI:** 10.3390/pathogens12121440

**Published:** 2023-12-12

**Authors:** Sylwia Jarzynka, Oliwia Makarewicz, Daniel Weiss, Anna Minkiewicz-Zochniak, Agnieszka Iwańska, Wojciech Skorupa, Marcin Padzik, Ewa Augustynowicz-Kopeć, Gabriela Olędzka

**Affiliations:** 1 Department of Medical Biology, Medical University of Warsaw, Litewska 14/16, 00-575 Warsaw, Poland; anna.minkiewicz@wum.edu.pl (A.M.-Z.); marcin.padzik@wum.edu.pl (M.P.); gabriela.oledzka@wum.edu.pl (G.O.); 2 Institute for Infectious Diseases and Infection Control, Jena University Hospital, 07747 Jena, Germany; oliwia.makarewicz@med.uni-jena.de (O.M.); daniel.weiss@med.uni-jena.de (D.W.); 3 Department of Microbiology, National Institute of Tuberculosis and Lung Diseases, Plocka 26, 01-138 Warsaw, Poland; a.iwanska@igichp.edu.pl (A.I.); e.kopec@igichp.edu.pl (E.A.-K.); 4 First Department of Lung Diseases, National Institute of Tuberculosis and Lung Diseases, Plocka 26, 01-138 Warsaw, Poland; w.skorupa@igichp.edu.pl

**Keywords:** cystic fibrosis, *Pseudomonas aeruginosa*, antimicrobial drug resistance

## Abstract

Background: *Pseudomonas aeruginosa* (PA) is one of the most predominant pathogens of lung infections, often causing exacerbations in adult patients with cystic fibrosis (CF). Materials and Methods: Microbiological characterization of 74 PA isolates and to evaluate the correlations between the bacterial features and 44 adult Polish CF cohort clinical parameters. Results: The most common variant in the CF transmembrane conductance regulator (*CFTR*) gene was F508del (76.3%), followed by 3849+10kbC>T (26.3%). A total of 39.4% of the PA isolates showed multiple resistances. In patients with parameters pointing to a decline in lung function, there was a statistically significant moderate correlation with β-lactam resistance and a weak correlation between hospital frequency and colistin resistance. The mucoidity did not correlate with the biofilm formation ability, which showed 41.9% of the isolates. Proteolytic activity, observed in 60.8% of the clinical isolates, was weakly associated with motility detected in 78.4% of the strains. The genetic profiles of the PA were highly heterogeneous, and a weak positive correlation was established between cluster group and biofilm formation. Conclusion: The findings suggest that there is a high variety in *P. aeruginosa* populations in adult CF patients. There is a need to monitor PA strains in groups of patients with cystic fibrosis, in particular, in terms of the occurrence of antibiotic resistance related to a decline in lung function.

## 1. Introduction

Cystic fibrosis (CF) is a life-threatening autosomal recessive genetic disease that affects a diverse population, with an estimated 100,000 patients globally [1]. CF is caused by mutations in the CF transmembrane conductance regulator (*CFTR*) gene on chromosome 7, which encodes a cAMP-regulated epithelial chloride channel expressed in many tissues [2]. A multitude of variants have been identified, with some being more prevalent than others and a local variation in prevalence. CF affects the epithelia in several organs, resulting in a complex, multisystem disease that involves the respiratory tract, exocrine pancreas, intestine, hepatobiliary system, male genital tract, and exocrine sweat glands. The lungs are usually the most affected part of the body. CF patients suffer from acute and chronic pulmonary infections caused by a variety of microorganisms. One of the most critical pathogens in adult patients is the opportunistic Gram-negative rod *Pseudomonas aeruginosa* [3,4]. Over the last few years, several studies have focused on *P. aeruginosa* infections in CF. Characteristically, children were the most widely studied group. This is explained by the fact that *P. aeruginosa* strains are acquired early in life [5]. According to the ECFS (European Cystic Fibrosis Society) 2020 registry, in Polish children with CF, the prevalence of chronic *P. aeruginosa* was 11.71%, and in adult CF patients the prevalence was 55.83% [6]. In the Polish CF study, a double mortality risk in patients with *P. aeruginosa* infection was observed [7]. Its capability to adhere to a variety of epithelial cells is believed to be the main cause of chronic infection, associated with a more rapid decline in lung function and increased morbidity and mortality [3,8]. The guidelines [9,10] recommend treatment of acute *P. aeruginosa* infections for up to 28 days with tobramycin solution for inhalation (TIS) and up to 3 months with a combination of nebulized colistimethate (a precursor of colistin) and oral ciprofloxacin. For chronic infections, besides the inhaled tobramycin formulation, inhaled aztreonam lysine and colistimethate are alternatively recommended for a period of 28 days. The dosing in CF is higher compared to non-CF lung infections, and the combination of two or even three antibiotics is recommended. All these results in a highly selective environment in the CF lung promoting resistance development and establishment. However, the specific conditions of the CF lung strongly contribute to the reduced efficiency of treatment. Particularly, the formation of a thick mucus layer in the epithelial cells in the CF airway and the formation of bacterial biofilms represent an extraordinary barrier for effective antimicrobial treatment. Biofilms are sessile matrix-embedded microbial communities whose formation is promoted by the thick, sticky mucus in CF patients. The matrix is produced by the microbes and is composed of oligosaccharides, proteins, and nucleic acids, which protect the bacteria against immune response and environmental stress [11]. Additionally, biofilm-embedded bacteria reduce their metabolic activity which renders most antibiotics ineffective. Thus, biofilms decrease the susceptibility to antibiotics, leading to treatment failure [11]. In particular, biofilms formed by the mucoid *P. aeruginosa* (producing the oligosaccharide alginate) are widespread in CF [12]. *P. aeruginosa* strains in early infection of the CF lung typically resemble those found in the environment, being non-mucoid, fast-growing, and relatively susceptible to antibiotics. Chronic *P. aeruginosa* infections promote the significant adaptation of the causative to the airway environment of the CF patient [13], such as a transition from the non-mucoid to the mucoid, alginate-overproducing phenotype [14]. It was noted that phenotypic and genotypic changes during chronic infection that enable *P. aeruginosa* to survive include the loss of motility and virulence (O-antigen components) and the emergence of multiantibiotic-resistant strains [15,16]. In Poland, there is not a national CF registry. Data from only a few clinical centers are reported to the ECFS Patient Registry. It is estimated that the total number of CF adult cases treated at the National Institute of Tuberculosis and Lung Diseases in Warsaw, which is a leading hospital for adult CF treatment in Poland, is about 100 patients. Because no decrease in mortality is observed in Polish CF patients, we would like to show the sighting relationships between microbiological agents and exacerbations of the infections of the adult CF Polish patients. The main goal of the study was the assessment of the effects of *Pseudomonas aeruginosa* features on the clinical condition of the Polish adult CF cohort.

## 2. Materials and Methods

### 2.1. Study Population

The cystic fibrosis adult patients, who had suffered from exacerbations due to *P. aeruginosa*, were treated between 2013 and 2018 at the First Department of Lung Diseases and CF Outpatients Care Clinic (CF-OOC) of the National Institute of Tuberculosis and Lung Diseases in Warsaw. This study was performed retrospectively on the collected data and microbiological material in accordance with the Helsinki Declaration and was approved by the institutional ethical committee under registration number KB-74/2019. 

### 2.2. Clinical Material

This study was conducted using clinical bacterial isolates from the sputum of adult CF patients who were expected to suffer from exacerbations due to *P. aeruginosa*. The sputum samples were taken within the microbiological control during hospitalization in infection exacerbation episodes or ambulatory follow-up treatments subsequent to the CF-OOC, two or more times in the period from 2013–2018.

### 2.3. Microbiological Methods of the Pseudomonas aeruginosa Isolates Characteristics

#### 2.3.1. Culture Conditions

Collections were performed in the Department of Microbiology at the National Institute of Tuberculosis and Lung Diseases in Warsaw. Phenotypic and genotypic tests were examined in the Department of Medical Biology of the Medical University of Warsaw. Cultures of *P. aeruginosa* were grown in Columbia blood agar with 5% sheep blood and MacConkey agar (all bioMérieux, Marcy-l’Étoile, France) at 37 °C for 24–48 h in an aerobic atmosphere. On selective chromid *P. aeruginosa* agar (bioMérieux, Marcy-l’Étoile, France), strains were incubated aerobically at 37 °C for 24 h. Identification of species was performed by phenotypical and morphological tests: enzyme cytochrome oxidase production by the *Pseudomonadaceae* family (Dryslide Oxidase, Becton Dickinson, Holdrege, NE, USA), pigmentation-pyocyanin detection on agar (Cetrimide Agar, Becton Dickinson, Holdrege, NE, USA), and including the use of Vitek2 Compact (bioMérieux, Marcy-l’Étoile, France). All strains were stored at −80 °C till handling. *P. aeruginosa* NCTC 6749 and *P. aeruginosa* PAO1 were used as reference strain.

#### 2.3.2. Antimicrobial Susceptibility Testing

Antimicrobial susceptibility testing (AST) was performed using disk diffusion for aminoglycosides (amikacin), β-lactams (ceftazidime, meropenem, tazobactam/piperacillin), fluoroquinolones (ciprofloxacin), E-test for aminoglycosides (tobramycin), and broth microdilution for polymyxin (colistin). All antimicrobials were purchased from Oxoid (Oxoid Deutschland GmbH, Wesel, Germany). Results were interpreted according to the European Committee of Antimicrobial Susceptibility Testing clinical EUCAST breakpoints from 2023 [17].

#### 2.3.3. Proteolytic Activity

Proteolytic activity was examined according to the method described by Kessler et al. (2014), Cheng et al. (2022), and Filloux et al. (2014) [18,19,20] but modified by using skim milk (Skim Milk Powder, Sigma-Aldrich, St. Louis, MO, USA) as a substrate. An overnight Luria Bertani (LB, Becton Dickinson, Holdrege, NE, USA) broth culture was spotted onto a 10% *v*/*v* skim milk agar plate and incubated at 37 °C for 24 h. The diameter of the transparent zone under and around the colony was measured and diameters > 5 mm were interpreted as positive for protease activity. The growth of the colony with a clear zone was described as a negative result.

#### 2.3.4. Mucoidy Ability

Mucoid isolates (slimy phenotype) were characterized by the presence of mucoid-producing cells on Pseudomonas Luria Bertani (LB, Becton Dickinson, Holdrege, NE, USA) with agar (Bacto Agar, Becton Dickinson, Holdrege, NE, USA) at 37 °C for 48 h. Strains with negative results were classified as non-mucoid.

#### 2.3.5. Biofilm Assays

Fresh *P. aeruginosa* suspensions were prepared in LB from overnight cultures. After incubation for approximately 8 h at 37 °C and orbital shaking, the stationary phase culture was diluted 1:100 in LB broth in triplicate in sterile 96-well polystyrene plates (Kartell S.p.a., Noviglio, Italy), which were incubated at 37 °C without shaking. After 24 h incubation, the microtiter plate wells were washed three times with 200 µL of sterile 1% phosphate-buffered saline (PBS, Thermo Fisher Scientific, Loughborough, Leicestershire, UK) and dried for 10 min at 60 °C. The biofilms were stained with 200 µL 0.2% crystal violet (Merck KGaA, Darmstadt, Germany) solution (*w*/*v* in deionized water) for 15 min at room temperature. The unbound crystal violet stain was removed, and the wells were washed gently three times with 200 µL of 1% PBS. The crystal violet in each well was solubilized by adding 200 µL of ethanol (96%, Chempur, Piekary Śląskie, Polska). The absorbance of each well was measured at 570 nm using the Synergy HTX multi-mode reader (Biotek Instruments, Inc., Winooski, VT, USA). The percentage change in biofilm biomass was analyzed by comparing the averaged values of the isolates to the control (no biofilm grown, broth samples without bacteria), and the classes were defined as follows: A_isolate_ ≤ A_control_-non-biofilm producer, A_control_ < A_isolate_ < 2 × A_control_-weak producer, 2 × A_control_ < A_isolate_ < 4 × A_control_–moderate, 4 × A_control_ < A_isolate_ and strong-producer. *P. aeruginosa* PAO1 was used as a positive biofilm reference strain.

#### 2.3.6. Motility Tests

Swarming motility was assayed on 0.2% agar M8 plates supplemented with 0.2% glucose, CAA 0.5% (Sigma-Aldrich, St. Louis, MO, USA), and 1 mM MgSO_4_ (Sigma-Aldrich, USA). Swimming motility was measured in 0.3% agar M8 minimal medium supplemented in the same manner as described by Cullen et al. (2015), Murray et al. (2010), and Lee et al. (2005) [21,22,23]. The presence of visible tendrils was proven (positivity), and the radial distance for the *P. aeruginosa* colonies was assessed after incubation at 37 °C for 24 h. To determine motility, the criteria that the growth area was ≥10% of the control PAO1 strain and the diameter of the growth area was >5 mm were used to consider the strain as motile. Strains showing a growth zone ≤ 5 mm were considered equal to the size of the initial inoculum, therefore such strains were scored as immobile.

#### 2.3.7. Genetic Analysis

Genotyping of the variations in the *CFTR* gene region was performed using the INNO-LiPA CFTR*iage* probe assay (Fujirebio, Tokyo, Japan) that simultaneously detects 88 according to the manufacturer’s recommendations in commercial diagnostic laboratories during cystic fibrosis proof. We analyzed retrospective data. Two patients were genotyped by sequencing by other institutions, where the respective patients were diagnosed with CF in the past and the genotype was provided in the patient’s records. For three patients with non-transparent medical records, no mutations were identified by the panel, or the patient refused genetic analysis.

### 2.4. Genetic Differences of the Clinical Isolates

Total DNA was extracted using the Genomic Mini kit (A&A Biotechnology, Gdańsk, Poland). Genotypic relatedness between the PA isolated was examined using the randomly amplified polymorphic DNA (RAPD) method using the primers Pa1 (5′AGGGGTCTTG 3′) and Pa2 (5′CTTCTTCAGCTCGACGCGACG 3′) (IBB PAN, Warsaw, Poland) as described previously [24]. Briefly, the RAPD-PCR reaction mixture (25 μL in total) contained 2.5 U of Taq DNA Polymerase, 200 µM dNTP mix, a 10 × reaction buffer supplemented with MgCl_2_ (all Merck KGaA), 10 µM each primer, and 2.0 μL of DNA solution (concentrations variable). The amplification was performed in the Mastercycler Personal (Eppendorf, Hamburg, Germany) as follows: 95 °C for 15 min, 44 cycles of denaturing at 94 °C for 30 s, annealing at 49 °C for 1 min, extension at 72 °C for 1 min and final extension at 72 °C for 10 min. All PCR reactions were performed in triplicates. No differences between the triplicates were visible. The final products of the reaction were separated by electrophoresis in a 2% agarose gel at 135 V for 2 h. The DNA Ladder 100–3000 bp (Blirt S.A., Gdańsk, Poland) was used to assess the size of the amplicons. Documentation was performed by ImageLab (Bio-Rad, Inc., Hercules, CA, USA). *P. aeruginosa* NCTC 6749 was used as a positive control and *Escherichia coli* ATCC 25922 and a non-DNA probe was used as negative controls.

The RAPD fingerprints of the best gels were analyzed by BioNumerics 7.6 (Applied Math NV, Sint-Martens-Latem, Belgium) applying the Dice similarity coefficient (0.5% optimization, 1% tolerance) and calculating the genetic distance to obtain a phylogenetic tree using the unweighted pair group method with arithmetic means (UPGMA) [25,26,27]. For the clustering, the Topscore UPGMA method was used. UMPGA calculates the average distance between clusters based on the assumption that all distance pairs are equal, building a hierarchical tree by successively merging the most similar clusters that reflect the nested relationships.

### 2.5. Statistical Analysis and Visualization

Descriptive statistics were performed on pseudonymized data using IBM SPSS Statistics 28.0.1 (IBM, Armonk, NY, USA). Using GraphPad Prism 9 (GraphPad Software, San Diego, CA, USA), the median, maximum and minimum values were obtained, and the normal distribution of the parameters was proven to apply the D’Agostino & Pearson omnibus normality test. Correlation analysis was performed using Spearman’s rank (*r_s_*) matrix as some parameters were non-metric. Graphics were performed using GraphPad Prism 9, CorelDRAW Graphics Suite 2021 (Corel Corporation, Ottawa, ON, Canada), and Microsoft 365 (Microsoft Corporation, Redmond, WA, USA).

## 3. Results

### 3.1. Clinical Parameters of the CF Patient Cohort

In this study, 44 CF patients were included. All required regular care in the CF-OCC, which included follow-up appointments and conservative treatment in case of exacerbations during chronic infection. Most patients were already being cared for at the clinic before the study. The study population was characterized by demographic and clinical data (Table 1). The study group consisted of 20 male and 24 female patients. The age at CF diagnosis was known for 39 patients and was on average 7.8 ± 11 years but was not normally distributed in the cohort. Thereby, 20.5% were diagnosed before reaching 1 year (minimum age was 1.2 months). On a cumulative basis, most diagnoses were obtained before reaching the third year (51.3%). Until the age of 10 years, 71.8% were CF diagnosed and 89.7% were until 18 years. In two cases, the diagnosis was made at the age of 20, and in two further extreme cases at the age of 46. In the male group, higher average values for the age of CF diagnosis, BMI parameter, and diabetes cases were observed. In turn, higher rates of the years under therapy, the number of hospitalizations frequency, and cases qualified for lung transplantation were observed in women. With regard to gender, no differences were observed in the incidence of pancreatic insufficiency and forced expiratory volume in 1 s or forced vital capacity indicators (data not shown).

The average age at first sampling within this study was 28.3 ± 7.1 years (minimum 18 years, maximum 53 years). The distribution did not pass the normality test because of the higher number of patients under 30 years of age. On average, the patients received medical care and were documented at the CF-OCC for 9.1 ± 4.6 years; this variable was distributed normally. During that time, the annual numbers of visits and hospitalizations varied strongly within the cohort, thus the means seem to be not representative. Most patients (86.4%) came to the clinic with an average number of visits times per year between 3.6 and 14.6. Two patients each presented on average 18.2, 24.3, or even 73 times per year. One of those two cases that presented with the highest frequency suffered from the FEV_1_ parameter (forced expiratory volume in 1 s) (33.5) and low BMI (body mass index) (16.2) and qualified for lung transplantation. The other patient was rather inconspicuous in relation to their physical condition, and the high visit frequency might have resulted from his first year at the CF-OOC and the optimization of the treatment. Due to their clinical condition, 7 (16.6%) patients qualified for lung transplantation, and two of these patients passed, both approximately two years after sampling at the age of 30 and 34, respectively.

The clinical parameters were distributed normally in the cohort. The FEV1 was on average 52.1 ± 22.2 L, the FVC was 71.6 ± 19.2 L, and the Tiffeneau-Pinelli index (FEV1/FVC) was 70.3% ± 15.9%. Exocrine pancreatic insufficiency was present in 72.7% of patients and diabetes in 34.1%. The age correlated well with the years under treatment at CF-OOC but also with the visit frequency. Weak but significant correlations between qualification for lung transplantation and BMI (*r_s_* = −0.42), hospitalization frequency (*r_s_* = 0.43), or the deterioration in lung function parameters (e.g., FEV1/FVC *r_s_* = −0.47) were determined. Age correlated also weakly in a positive manner with BMI (*r_s_* = 0.32) and diabetes (*r_s_* = 0.31). The decline in lung function correlated weakly with qualification to lung transplantation (*r_s_* = −0.47), hospitalization frequency (*r_s_* = −0.35), BMI (*r_s_* = 0.38), and diabetes (*r_s_* = −0.36) (Supplementary Material, Table S1).

### 3.2. Variants Related to CF

**Figure 1:**
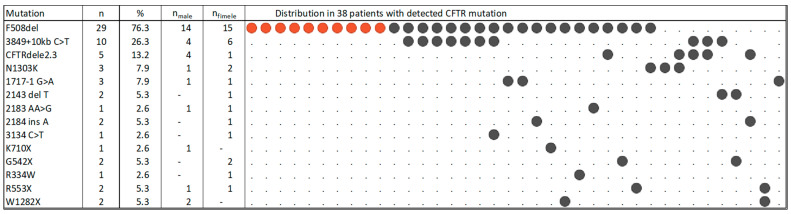
Identified variants (variant legacy names) in CF-relevant target genes and their distribution in the patients. Note: Dark grey circles indicate a variant only in one chromosome, while red circles indicate an F508del homozygote variant (on both chromosomes). Patients without genetic characterization were not included in this figure.

Genotyping was provided for 38 (86.4%) of the study participants. Thereby, genetic characterization was available for 20 female (83.3%) but only 18 male (45%) patients. The most prevalent variant in those was the deletion of phenylalanine at position 508 (F508del) which occurred in 76.3% of patients with 1/3 (26.3%) of those being homozygote and without any other detected variant in the used panel (Figure 1). Out of the patients with an F508del variant, 15 were female (4 being homozygote), and 14 were male (6 being homozygote). Except for three patients, the residual patients carried two different identified variants (compound heterozygote genotype), of which 17 patients were heterozygote for F508del accompanied by one other variant, with variant 3849+10kbC>T being the most frequent. In one patient each, only the heterozygote F508del variant or N1303K or 1717 were identified. One patient carried the F508del variant accompanied by two further variants (3849+10kbC>T and 3134 C>T with so far unknown effect on the CF pathogenicity [28]). The overall frequency of 3849+10kbC>T was 26.3% (*n* = 10) in all positively tested patients. Furthermore, the five patients being compound heterozygotes for 3849+10kbC>T with F508del (13.2%), three other patients were compound heterozygotes with another variant (two with *CFTR*dele2,3, one with 2143 del T). With 13.2% (*n* = 5), variant *CFTR*dele2,3 was the third common variant followed by N1303K and 1717-1G>A (both with 7.9%, *n* = 3). The other variants were found in one (2.6%) or two (5.3%) cases only.

The distribution of the variants was slightly different in male and female patients in the frequency of the homozygote F508del and the *CFTR*dele2,3 variants, which occurred more frequently in men vs. women (with 33% vs. 20% and 22% vs. 5%, respectively). Due to the low abundance of the other variants, correlations to clinical parameters were only possible for the F508del variant. This correlated weakly with exocrine pancreatic insufficiency (*r_s_* = 0.39) (Supplementary Material, Table S1).

### 3.3. Microbiological Properties of the P. aeruginosa Isolates

In total, 74 *P. aeruginosa* strains were isolated from the sputum of the cohort. Thereby, from 24 patients only one isolate was collected at one visit. In addition to the first visit, follow-up isolates were collected for 20 patients, so there are two independent isolates from two visits for 13 patients, three isolates from three visits for three patients, and four isolates from four visits for one patient. There are four isolates from three visits for two patients, and three isolates from two visits for one patient two colonies of different morphologies were selected for analysis. In total, 33.8% of isolates showed mucoid phenotype, and 41.9% formed biofilms in vitro (Table 2). Protease activity was found in 60.8% of isolates and 78.4% of the isolates showed increased motility due to swimming and swarming (Table 2). Interestingly, the mucoidity did not correlate with the biofilm formation ability. Swarming weakly correlated (*r_s_* ~ 0.4) with swimming and proteolytic activity (Supplementary Material, Table S2). The growth of the colony with positive and negative results to phenotypic properties was shown in the figure Supplementary Material, Figure S1.

Susceptibility to the seven tested and commonly used antimicrobials in CF was found in 39.2% of the isolates. Resistance against at least one compound was found in 21.6% of the isolates, while 39.4% showed multiple resistances with 12.2% being resistant against five compounds; one isolate was resistant to all tested antimicrobials including colistin (Table 2). The distribution of the inhibition zone diameters of piperacillin/tazobactam, ceftazidime, meropenem, ciprofloxacin, and amikacin, and the minimal inhibitory concentration of tobramycin and colistin were shown in Supplementary Material, Figures S2 and S3, respectively.

The number of isolates was too low to draw conclusions on specific resistance patterns, but resistance frequencies against a specific drug were generally higher in non-mucoid isolates (Table 3). The highest resistance rates were observed for amikacin (37.8%) followed by ciprofloxacin (36.5%). Approximately 1/3 of the isolates were resistant to ceftazidime (32.4%), piperacillin/tazobactam (29.7%), and tobramycin (27.3%) and 19 isolates (25.7%) were carbapenem-resistant. Within the β-lactam resistant isolates, only 7 (9.5%) could be solely categorized as extended-spectrum β-lactamase (ESBL) phenotype, and 3 (4.1%) were solely resistant to the β-lactamase inhibitor (BLI) tazobactam, but 9 isolates (12.2%) were resistant to BLI, ceftazidime, meropenem and ciprofloxacin, classified as 4MRGN in Germany. Thus, not surprisingly, the resistances correlated all moderate (*r_s_* > 0.5) to strong (*r_s_* > 0.7) to each other, except for colistin resistance, which was only present in four strains. There was also a weak correlation (*r_s_* > 0.3) between the mucoidity and susceptibility to aminoglycoside as well as a lower number of present resistances (Supplementary Material, Table S2).

### 3.4. Genotypic Differences of Clinical P. aeruginosa Isolates

**Figure 2:**
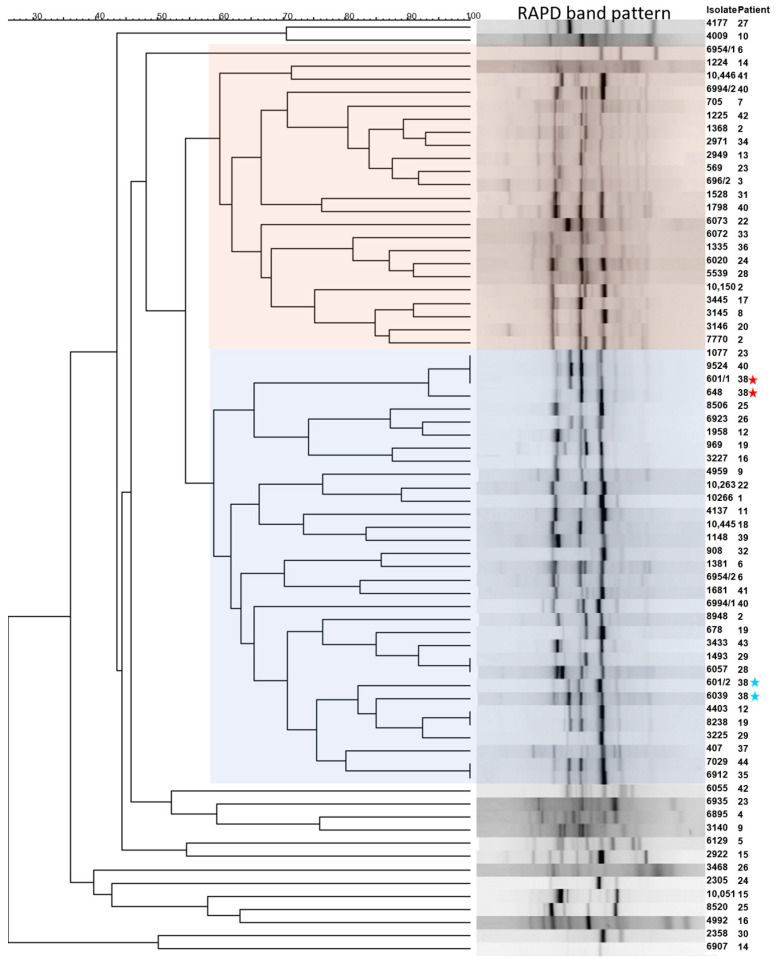
The dendrogram of the *P. aeruginosa* isolates was calculated based on the RAPD. Note: The evaluation of the band pattern by applying the Dice algorithm and UPGMA grouping method. The isolates’ numbers and the corresponding randomized patient numbers are indicated on the right site. Two bigger clusters were colored orange and blue. (Exemplary: red asterisk indicates two isolates obtained at one visit, the blue asterisks the corresponding two isolates from other visits of patient 38).

The RAPD yielded measurable signals in 71 isolates corresponding to 41 patients (Supplementary Material, Figures S4 and S5). There was observed a high wide genetic variability of *P. aeruginosa* isolates, suggesting genotypic differences. Two bigger RAPD groups, each with approximately 60% similarity were observed. One of the clusters (I, highlighted in orange in Figure 2) contained 22 (31.0%) of the analyzed isolates, and the other (II, highlighted in blue in Figure 2) was 33 (46.5%). The other 16 (22.5%) strains formed smaller groups with similarities between 60% and 75% but did not merge within the two clusters. In general, the RAPD-band matching resulted in a strongly diverse phylogenetic pattern. It was particularly striking that almost no RAPD similarities were found within the isolates obtained from one patient. In three cases where two isolates exist from one visit, only in one case, the isolates were in a close RAPD neighborhood, which suggests relationships between them (Figure 2, indicated by a red asterisk). They also showed similar resistance patterns, but one was mucoid and the other non-mucoid, but a clonal relation is likely for this pair. In the same patient, the other two isolates obtained from other visits (Figure 2, indicated by a blue asterisk) belonged to the same RAPD group and showed a lower distance to each other in their RAPD pattern which might also indicate clonal linage. In four cases, the pattern of two (three cases) or three (one case) isolates showed identical band patterns, but all these isolates were from different unrelated patients thus the RAPD similarity can be purely accidental.

**Figure 3:**
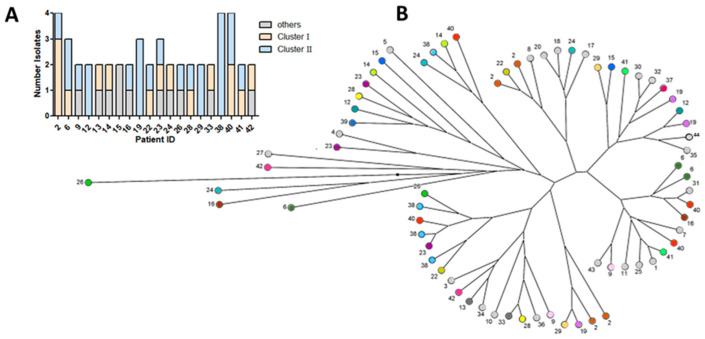
Cluster analysis of the genetic relationship of 49 *Pseudomonas aeruginosa* isolated from 20 CF patients (**A**) and 71 *P. aeruginosa* isolates identified in 41 patients with RAPD-pattern (**B**). The dots ending the RAPD lineages represent the randomized numbers of the patients. Patients with only one isolate were indicated in light grey, and patients with only one isolate analyzed due to missing RAPD-pattern of the other(s) isolate(s) are shown in dark grey. Colored dots indicate patients (color-matched) with more than one analyzed isolate.

It was also striking that the strains within one patient often belonged to different clusters or to the non-clustering groups (Figure 3A). Based on the phylogenetic diagram, a cluster analysis was performed using patient numbers as an indicator to visualize the variability of the *P. aeruginosa* isolates in the patients (Figure 3B). Additionally, clonality was investigated only on the repeated 49 isolates from 20 patients considering the phylogenetic grouping (Figure 3A) and phylogenetic properties, including resistogram pattern (Supplementary Material, Figure S6). Comparing only the resistance pattern, more similarities per patient were found between the isolates, but only in a few patients was the RAPD group suggesting similarity. The RAPD group showed a weak correlation (*r_s_* > 0.3) only with the ability of biofilm formation, thereby the phylogenetic group defined as ‘group II’ builds stronger biofilms.

### 3.5. Correlation of Patients and Pathogen Characteristics

The characteristics of both the patients and pathogen were performed only using the isolate of the first study sampling. Thus, some parameters of the pathogens were excluded: colistin resistance due to low abundance and RAPD group due to high variability. All results are presented in Table 4. In general, the characteristics of the pathogens did not correlate much with the patient’s pathophysiology. There was no correlation found between the ability to form biofilms, mucoidal phenotype, protease activity or motility, and the clinical parameters, such as hospitalization frequency, FEV_1_ /FVC, and qualification for lung transplantation. The patient’s age correlated negatively but weakly to moderate (*r_s_* < 0.5) with the number of determined resistances in the corresponding isolate and the 4MRGN phenotype. There was also a weak negative correlation (*r_s_* ~ −0.3) between the number of resistances per isolate and the years under treatment but a positive weak correlation to visit frequency. The meropenem resistance also correlated moderately (*r_s_* = 0.5) with reduced BMI. The hospitalization frequency correlates weakly to moderately (*r_s_* values 0.33 to 0.54) with all investigated resistances. Worsening in lung function correlated weakly (*r_s_* values 0.34 to 0.41) with resistance against all tested β-lactams but not the other antimicrobials.

## 4. Discussion

The objective of the current study was to analyze a cohort of 44 adult cystic fibrosis patients who had a documented history of *P. aeruginosa* infection. We characterized and correlated the clinical characteristics, demographics, as well as microbiological diversity of 74 *P. aeruginosa* strains collected from these patients. According to the Cystic Fibrosis Foundation, 75 percent of people with CF are diagnosed in early childhood until the age of two thanks to the CF newborn screening (CF-NBS) programs that are already routine in many countries. In the analyzed Polish CF-cohort, only 51.3% were diagnosed until the age of two, and five patients were diagnosed at the age ≥ 18 years, which corresponds to a prevalence of 11.4%. In Poland, the CF-NBS program started in 2006 in some regions and was implemented nationwide until mid-2009 [30]. All patients included in the present study were born before 2001, and thus were diagnosed before this measure. That explains the high rate of late diagnosis. It must be mentioned, however, that despite the CF-NBS, still, some patients with unknown CF-predisposition and mild symptoms are still unrecognized until adolescence or even adult age [31]. Based on the national CF register in Italian, where the newborn screening was implemented in 2016–2017, Padoan et al. described the prevalence of the diagnosis at age ≥ 18 years of 18.2% in 2012 and an incidence of 12.5% in 2018 [32]. They could correlate the late age of diagnosis to a low index of pancreatic function (25%) and *P. aeruginosa* infections (17%). In this view, the observed prevalence in this study cohort remains acceptable.

There is still no active cystic fibrosis register in Poland and according to a study by Rachel et al., the quality of healthcare provided to CF patients in Poland does not meet all European standards [33]. Polish cystic fibrosis patient databases from only 13 individual hospital centers contribute to the EUROCARE CF (European Centers of Reference Network for Cystic Fibrosis) and ECFS (European Cystic Fibrosis Society) registry. According to the ECFS patient registry from 2020, the proportion of living Polish CF patients was 66.74% (*n* = 889) children (<18 years) and 33.26% (*n* = 443) adults (≥18 years) [6]. Therefore, it is likely that many adults with CF in the Polish population remain undiagnosed until severe symptoms as reflected in two extreme cases occurred at the age of 46. These two oldest patients in the study cohort were siblings bearing identical variants 3849+10kbC>T and CFTRdele2,3. The 3849+10kbC>T variant results in a mild course of CF disease even if combined with heterozygote F508del [34]. The other variant, CFTRdele2,3(21kb) leads to a loss of exons 2 and 3 in the mRNA and a frameshift in exon 4 resulting in a stop codon and truncated CFTR protein [35]. This variant results in severe symptoms, including exocrine pancreatic insufficiency when occurring as compound heterozygote with F508del and thus has been associated with an early diagnosis. As these two patients were diagnosed so late and only one suffers from pancreatic insufficiency, the combination with 3849+10kb C>T seems to result in mild symptoms. Patients with this variant are usually diagnosed early and show a wide range of mild to severe clinical symptoms in adulthood: from normal sweat chloride concentration, and normal pancreatic and liver function but with pulmonary damages [36,37,38], to severe manifestations like acute pancreatitis, chronic diarrhea, hyper-inflammation in pneumoniae or bronchiectasis [38].

The CFTRdele2,3 variant is generally more common in Central and East Europe [35]. In 2000, an average frequency of 1.5 was reported for Polish and German cohorts; for Czech and Russian cohorts even higher (6.4 and 5.8, respectively) [35]. In our study, this variant was found in 12.8% of the patients. This could be a result of improved diagnostics, which implemented this allelic variant in test panels after evidence has been gained that this variant also contributes to infertility in male CF patients [35]. Dörk et al. showed that male patients being heterozygous for CFTRdele2,3 with other variants were initially diagnosed with infertility. Considering the possibility that non-F508del heterozygotes of CFTRdele2,3 might come with milder symptoms, this might be a reason for the observed higher frequency of CFTRdele2,3 in male patients. However, this is only a hypothesis as we did not investigate the initial hospital history/diagnosis that is often missing in the patient’s records.

The most abundant CFTR variant with 54.54% described for Polish CF-patients Ziętkiewicz et al. was F508del, followed by CFTRdele2,3 (4.47%) and 3849+10kbC>T (3.93%) [39]. In the present study, the frequencies of these variants were generally higher, as well as for the variant 2183AA>G (10.3%) detected by Ziętkiewicz et al. in 1.02% and recorded by ECSF registry in only 0.71% of CF patients within 39 countries [6]. This may be a local anomaly, but it is more likely that the pre-selection of the cohort by an expected exacerbation with suspected *P. aeruginosa* infection biases the observed prevalence. High frequencies of these variants were reported also in Armenia (2183AA>G variant in 10.0%), in Belarus (CFTRdele2,3 in 10.9%), and in Lithuania (3849+10kbC>T in 10.3%) [6]. These variants are known to be related to severe symptoms and it seems that they increase the risk of *P. aeruginosa* infections. In two unrelated CF patients with each bearing only one (but different) identified variant in the *CFTR* gene, most likely there were other variants present but were not detected by the chosen panels, as carriers of a single heterozygous *CFTR* gene variant (CF carriers) still produce enough of the CFTR protein to be considered healthy [40]. Life expectancy in the Polish CF population is increasing, but the number of deaths remains high. Especially in the 30–40 age group the risk of mortality increases. The most relevant risk factors identified by Durda-Masny *et at.* were infections with multi-resistant *P. aeruginosa* and F508del homozygote genotype, but also an FEV_1_ <  40% and BMI <  18.5 [7]. In our cohort, two patients at the age of 28, and 32 passed away waiting for lung transplantation due to worsening in lung function (FEV_1_ ≤  21.5%), a low BMI (≤16.5), and pancreatic insufficiency, but only for one the genotype was determined and was F508del heterozygote. The *P. aeruginosa* isolates obtained from those patients were susceptible to all β-lactams but were resistant to colistin and aminoglycosides (tobramycin or amikacin, respectively). In one of those patients, the isolate was additionally resistant to ciprofloxacin. Particularly inhaled aminoglycoside antibiotics strongly improve lung function in CF patients, and both reduce the bacterial load [41,42]. Thus, the low FEV_1_ of both patients might be also related to the treatment difficulties. However, we did not examine the treatment regiments of the patients and thus this statement is purely hypothetical.

The highly selective CF-lung environment directly translates to the observed exceptionally high resistance rates for different antimicrobials [29], which is in line with a recent meta-analysis of Bonyadi, et al. (based on 122 articles published until 2021) reporting on resistance prevalence in *P. aeruginosa* isolates from CF patients for two time periods (1979–2010 and 2011–2021) and in different regions. The resistance rates for the antibiotics tested in our study match quite well with this meta-analysis for ceftazidime, colistin, and amikacin, but were on the upper CI limit or slightly above for the other antimicrobials (Table 3). This is in line with the generally higher resistance rates in Poland and other East European countries compared to West and Middle European countries [43]. Of concern, is the high rate of isolates being resistant to 5 (12.2%) or more (10.9%) antimicrobials, which might result in treatment failure.

In many studies, mucoid phenotypes of *P. aeruginosa* were described as strong biofilm producers and the main agents of chronic infections in CF patients by multi-resistant *P. aeruginosa* strain [44,45,46,47]. Malhotra et al. reported that *P. aeruginosa* mucoid and non-mucoid variants spread in the CF lungs, but the non-mucoid phenotypes are cultured from sputum more often at the beginning of infection [47]. In our study, there was a weak correlation between multidrug resistance and non-mucoid phenotype. This might be biased by the generally higher prevalence of non-mucoidal strains in CF patients which is in agreement with the study of Adkin et al. [48]. However, the acquisition of the resistance particularly to colistin, tobramycin, meropenem, and ciprofloxacin by non-mucoid *P. aeruginosa* strains was previously related to suppressive antimicrobial therapy in chronically infected CF patients by Fernandez-Barat et al. [49]. A weak correlation between colistin, tobramycin, and ciprofloxacin found in our study was confirmed by other authors who characterized the additivity effect of ciprofloxacin/colistin and tobramycin/colistin combined therapy [50,51]. Our observation that the type of colony (mucoid or non-mucoid) does not influence biofilm formation ability was also in agreement with this study.

To study the genetic diversity of the *P. aeruginosa* isolates from the CF cohort, we decided to perform RAPD, which is a widely used, rapid, cost-effective, and suitable method [25,26,27]. It does not have the deepness of sequencing, but it provides enough information for these types of analyses. The phenotypic correlation of bacterial properties revealed a weak correlation between the RAPD group and the biofilm formation indicating that one of the lineages might be related to stronger biofilm formation. It might be interesting to further analyze this observation and to figure out which genetic factors are associated with increased biofilm formation ability. These might help to find some new targets or strategies for treatment.

However, the collected isolates showed very high variability. There was almost no RAPD-clonality of the strains even in the same patient. In three patient samples, two *P. aeruginosa* colonies at each visit were further analyzed for visible differences in colony morphology, but only in one case clonality was likely due to identical RAPD group, resistance pattern, and protease activity and motility, however, characteristics like biofilm strength and mucoidity were different. This could be an indication that the population of *P. aeruginosa* can differ significantly in its morphology despite being genetically similar, but we did not further investigate the underlying resistance genes in this study. Specific antimicrobial resistance genes encoded on resistance plasmid can transmit between the different *P. aeruginosa* subspecies. This can result in a synchronized resistance pattern in a lung population and is consistent with our observation that the resistance patterns were more similar than the RAPD groups within one patient. On the other hand, the development of resistance in *P. aeruginosa* (e.g., to β-lactams or fluoroquinolones) can also be due to secondary mechanisms such as increased efflux activity and/or porin loss [52]. A recently published study demonstrated that stochastic expression favors such mechanisms through regulatory mutations [53], which can lead to heterogeneity in phenotypic resistance. However, to prove these considerations a higher number of cases and longitudinal samples must be evaluated by genome sequencing.

In the future, intensive time-dependent genetic analysis of CF patients will show, how the lung microbiome changes and how modern therapies and extended life expectancy influence it. Recent genetic studies demonstrated the potential of sequencing in CF diagnostics [54] verifying the variability of *P. aeruginosa* population in CF lung. This is in line with our results, which show that the CF lung is colonized by a highly variable *P. aeruginosa* community, and in only limited cases those are descent from one ancestor as we did not find many such cases even applying only 60% similarity for RAPD-grouping.

Based on clonality analysis, it has been previously shown that the paranasal sinuses and oral cavity are possible reservoirs for lung infection in CF patients [55,56]. Those are frequently colonized or infected by environmental *P. aeruginosa* strains, which can reinfect the lower airways and the lung resulting in the observed variability in the isolates [55,57]. The large genetic diversity of *P. aeruginosa* was described in various cohorts [52,54,58,59,60] and may be related to mutations that occur during long-term colonization or persistent infection and antibiotic therapy [59]. It has been shown that some clonal CF *P. aeruginosa* complexes are highly conserved at the sequence level (<1% nucleotide divergence) [53,61]. The genomes of the underlying *P. aeruginosa* show little variability in the early stage of a chronic lung infection, but with the duration of the chronic course, single nucleotide mutations and genetic inversions in accessory and housekeeping genes result in diversification. On the other hand, previous studies showed that CF patients can be simultaneously colonized by several different strains that might be residents of the pulmonary tract microbiome prior to an exacerbation [62,63]. All these findings emphasize that antimicrobial resistance testing based solely on a single isolate may lead to ineffective treatment against other coexisting subspecies within the cystic fibrosis (CF) lung. As a result, the current guidelines should be re-evaluated to explore how CF diagnostics can be enhanced in the future.

Our study has certain limitations. A lack of comparison of *P. aeruginosa* diversity in other non-cystic fibrosis samples collected from the same period and region. The statistical analysis does not include the correlation between treatment regimens of the patients and their lung functionality features, as well as in subsequent exacerbations. It is important to confirm the clonal relationship by using a more advanced technology, such as Illumina or nanopore sequencing followed by gMLST typing for representative isolates from the same patients and isolates from chronic and intermittent infection.

Nonetheless, our study also possesses notable strengths, including comprehensive phenotyping of *P. aeruginosa* isolates, thorough investigation of exacerbation and etiology, and correlation with patients’ pathophysiology to better understand the *P. aeruginosa* subtypes associated with lung infection exacerbations in Polish adult CF cohort.

## 5. Conclusions

The results of the various correlation analyses performed to elucidate the interactions between clinical parameters and bacterial characteristics are inclined to continue research on a large number of isolates and other microorganisms from Polish cystic fibrosis patients. Our findings suggest that pathogen characteristics have minimal influence on CF pathophysiology; however, resistance to β-lactam antibiotics (specifically piperacillin/tazobactam and ceftazidime) may serve as indicators of an anticipated decline in lung function and should therefore be closely monitored.

## Figures and Tables

**Table 1:** Clinical characteristics of the study population.

Total CF Population, n	44
Female, n (%)	24 (54.5)
Male, n (%)	20 (45.5)
Pancreatic insufficiency, n (%)	32 (72.7)
Diabetes, n (%)	15 (34.1)
Qualification for lung transplantation, n (%)	7 (15.9)
Genetic characterization of the CFTR (%)	38 (86.4)
	average ± SD (median, min–max)
Age in the study period average	28.3 ± 7.1 (27, 18–53)
Age at CF diagnosis	7.8 ± 11 (2, 0.01–46)
Years under therapy	9.1 ± 4.6 (8.5, 1–20)
Hospitalization frequency [n/a]	1.1 ± 1.2 (0.9, 0–4.8)
Visits frequency at CF-OOC [n/a]	12.7 ± 14.2 (8.6, 3.7–73)
BMI [kg/m^2^]	19.8 ± 3.0 (19.9, 13.5–28)
FEV_1_ [L]	52.1 ± 22.2 (48.2, 14.7–99.8)
FVC average [L]	71.6 ± 19.2 (69.2, 28.5–116.9)
FEV_1_/FVC [%]	70.3 ± 15.9 (72.2, 37.9–100)

SD = standard deviation, CFTR = cystic fibrosis transmembrane conductance regulator, CF-OOC = CF Outpatients Care Clinic, BMI = body mass index, FEV1 = forced expiratory volume in 1 s, FVC = forced vital capacity.

**Table 2:** Characteristics of the *P. aeruginosa* isolates.

Phenotype	Isolates with Respective Phenotype*,* % (n)
Mucoidal phenotype	33.8 (25)
Protease activity	60.8 (45)
Motility (total)	78.4 (58)
Swimming	67.6 (50)
Swarming	62.2 (46)
Biofilm formation (in total)	41.9 (31)
Weak biofilm producers	16.2 (12)
Moderate biofilm producers	8.1 (6)
Strong biofilm producers	17.6 (13)
Number of resistances per isolate against the tested compounds:	
0	39.2 (29)
1	21.6 (16)
2	6.8 (5)
3	5.4 (4)
4	4.1 (3)
5	12.2 (9)
6	9.5 (7)
7	1.4 (1)

**Table 3:** Frequency of resistant phenotypes against commonly used antibiotics in mucoid and non-mucoid *P. aeruginosa* isolates.

Resistances Against	In the Study Cohort, % (n)	Weighted Pooled Resistance Prevalence 2011–2021 [29], % (95% CI)	Non-Mucoid, % (n)	Mucoid, % (n)
Piperazillin/tazobactam	29.7 (22)	19 (15–24)	24.3 (18)	5.4 (4)
Ceftazidime	32.4 (24)	36 (29–44)	25.7 (19)	6.8 (5)
Meropenem *	25.7 (19)	19 (15–24)	21.6 (16)	4.1 (3)
Ciprofloxacin **	36.5 (27)	28 (33–36)	27 (20)	9.5 (7)
Colistin ^#^	5.6 (4)	5 (2–8)	5.4 (4)	0
Tobramycin	27.3 (20)	22 (17–28)	24.3 (18)	2.7 (2)
Amikacin ***	37.8 (28)	38 (24–58)	32.4 (24)	5.4 (4)

CI = Confidence interval, n = amount, * three isolates were intermediate for meropenem, ** two isolates were intermediate for ciprofloxacin, *** one isolate was intermediate for amikacin, ^#^ not tested in 2 isolates.

**Table 4:** Correlation analysis of the clinical parameters of the CF-cohort and the *P. aeruginosa* properties (*n* = 44) at first sampling expressed as Spearman’s rank coefficients (*r_s_*, confidence interval 95%).

	Biofilm Ability	Mucoid	Proteolysis	Swimming	Swarming	No. Resistances	4MRGN	PIP/TAZ	CAZ	MEM	CIP	TB	AK
Age	0.03	0.10	−0.12	−0.12	−0.14	−0.43	−0.37	−0.03	−0.03	−0.18	−0.29	−0.16	−0.06
Sex	0.10	0.08	−0.22	−0.13	−0.08	0.08	−0.20	−0.22	−0.02	−0.04	−0.27	−0.07	−0.11
BMI	−0.03	0.02	−0.11	0.04	0.13	−0.03	−0.26	−0.18	−0.15	−0.50	−0.20	−0.18	−0.33
AD	−0.12	0.28	−0.12	0.14	−0.04	−0.25	−0.16	−0.10	−0.06	−0.08	0.03	−0.10	−0.12
YT	0.11	0.02	−0.11	−0.23	−0.28	−0.31	−0.25	0.01	0.04	−0.04	−0.06	−0.01	0.14
HF	0.03	0.03	−0.08	−0.40	−0.24	0.08	0.35	0.38	0.54	0.53	0.32	0.46	0.35
FEV_1_	0.06	0.06	−0.17	0.20	0.19	0.07	−0.13	−0.41	−0.35	−0.34	−0.06	−0.04	−0.12
FVC	0.02	−0.05	−0.10	0.17	0.04	−0.04	−0.07	−0.25	−0.28	−0.28	0.02	0.00	−0.01
FEV_1_/ FVC	0.08	0.16	−0.25	0.19	0.22	0.14	−0.11	−0.41	−0.32	−0.30	−0.10	−0.04	−0.15
LT	0.08	0.19	0.09	−0.21	−0.19	−0.01	0.12	0.13	0.21	0.30	0.09	0.01	0.17
Diabetes	−0.12	−0.14	−0.12	0.11	0.05	−0.27	0.03	0.16	0.09	0.07	−0.05	−0.01	0.12
PI	−0.17	0.04	−0.17	−0.12	0.07	0.14	0.16	0.06	0.23	0.26	−0.04	0.26	0.17
VF	−0.11	−0.02	0.11	0.23	0.28	0.31	0.25	−0.01	−0.04	0.04	0.06	0.01	−0.14

BMI = body mass index, AD = age at diagnosis, YT = years under treatment, HF = hospitalizations frequency, FEV_1_ = forced expiratory volume in 1 s, FVC = forced vital capacity, QLT = qualified for lung transplantation, PI = pancreatic insufficiency, VF = visit frequency, 4MRGN = resistant against tazobactam/piperacillin, ceftazidime, meropenem and ciprofloxacin, PIP/TAZ = piperacillin/tazobactam, CAZ = ceftazidime, MEM = meropenem, CIP = ciprofloxacin, CL = colistin, TB = tobramycin, AK = amikacin, *p*-values: dark grey ≤ 0.001 ≥ grey ≤ 0.01 ≥ light grey ≤ 0.05. The correlation significance (two-tailed) was assumed at level ≤ 0.05 (underlined in color). The following assumptions were applied for the correlation: weak *r_s_* > 0.3, moderate *r_s_* > 0.5, strong *r_s_* > 0.7, very strong *r_s_* > 0.9.

## Data Availability

More details information of the study data is available upon request to sylwia.jarzynka@wum.edu.pl.

## Supplementary Materials

The following supporting information can be downloaded at: https://www.mdpi.com/article/10.3390/pathogens12121440/s1, Figure S1. Examples of phenotypes of (A) proteolytic activity, (B) swarming motility, and (C) swimming motility; Figure S2. Distribution of the inhibition zone diameters of the CF-isolates assessed by antimicrobial susceptibility testing by disk diffusion method and the protease activity measured as the transparent zone on skim milk agar; Figure S3. Distribution of the minimal inhibitory concentration (MIC in mg/L) of the CF-isolates assessed by antimicrobial susceptibility testing by E-test strips (Tobramycin) and broth microdilution (Colistin). Table S1. Correlation analysis of the clinical parameters of the CF-cohort (*n* = 44) at first sampling expressed as Spearman’s rank coefficients (confidence interval 95%); Table S2. Correlation analysis of the *Pseudomonas aeruginosa* properties (*n* = 74) at first sampling from the CF-patient cohort expresses Spearman’s rank coefficients (*r_s_*, confidence interval 95%). Distribution of the minimal inhibitory concentration (MIC in mg/L) of the CF-isolates assessed by antimicrobial susceptibility testing by E-test strips (Tobramycin) and broth microdilution (Colistin); Figure S4. RAPD gels; Figure S5. RAPD gels; Figure S6. Visualization of the phenotypic characteristics of the *P. aeruginosa* isolates in 20 patients with multiple samples.
