# Supplementary material for: The Impact of Pseudomonas aeruginosa Infection in Adult Cystic Fibrosis Patients—A Single Polish Centre Study
Source: Pathogens. 2023 Dec 12;12(12):1440. doi: 10.3390/pathogens12121440 (PMC10748198; doi:10.3390/pathogens12121440)
Supplement: Supplementary file 1 [file pathogens-12-01440-s001.zip › pathogens-2748490-SI.pdf]

Supplementary Material

The impact of *Pseudomonas aeruginosa* infection in adult cystic fibrosis patients – a single Polish centre study

Sylwia Jarzynka [1,*,#], Oliwia Makarewicz [2,#], Daniel Weiss [2], Anna Minkiewicz-Zochniak [1], Agnieszka Iwanska [3], Wojciech Skorupa [4], Marcin Padzik [1], Ewa Augustynowicz-Kopec [3], Gabriela Oledzka [1]

[1]Department of Medical Biology, Medical University of Warsaw, Poland (sylwia.jarzynka@wum.edu.pl, anna.minkiewicz@wum.edu.pl, marcin.padzik@wum.edu.pl, gabriela.oledzka@wum.edu.pl)
[2]Institute for Infectious Diseases and Infection Control, Jena University Hospital, Jena, Germany (oliwia.makarewicz@med.uni-jena.de, daniel.weiss@med.uni-jena.de)
[3]Department of Microbiology, National Institute of Tuberculosis and Lung Diseases, Warsaw, Poland (a.iwanska@igichp.edu.pl, e.kopec@igichp.edu.pl)
[4]First Department of Lung Diseases, National Institute of Tuberculosis and Lung Diseases, Warsaw, Poland (w.skorupa@igichp.edu.pl)
*Correspondence: Sylwia Jarzynka, Litewska 14/16, 00-575 Warsaw, Poland, sylwia.jarzynka@wum.edu.pl, +48 22 1169250 (SJA)
[#]These authors contributed equally to this work

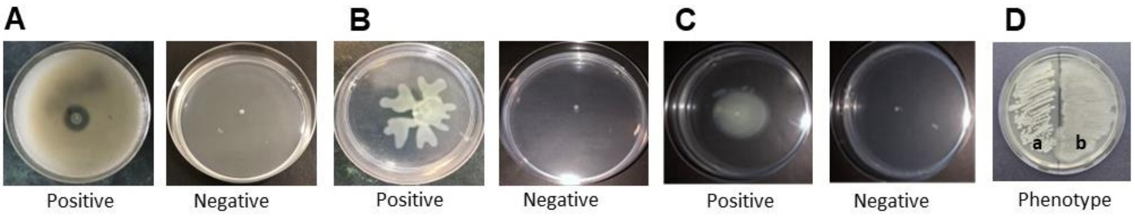

Figure S1. Examples of phenotypes of (A) proteolytic activity, (B) swarming motility, (C) swimming motility, and (D) non-mucoid (a) and mucoid (b) phenotype.

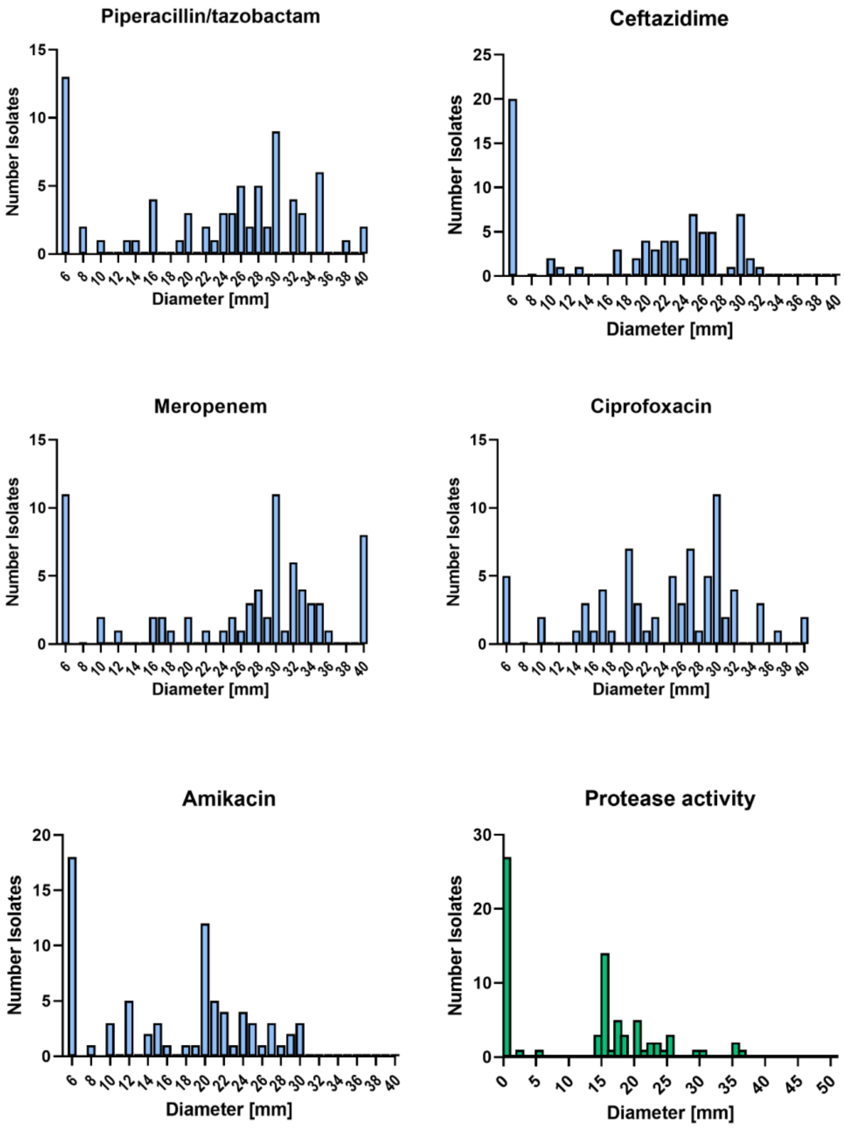

Figure S2. Distribution of the inhibition zone diameters of the CF-isolates assessed by antimicrobial susceptibilty testing by disk diffusion method and the proteaese activity measured as the transparent zone on skim milk agar.

The clinical antimicrobial susceptibility breakpoints were applied according to the recent EUCAST guidelines (https://www.eucast.org/clinical_breakpoints) as follows: piperazillin / tazobactam R < 18 mm, ceftazidime R < 17 mm, meropenem R < 14 mm, ciprofloxacin R < 26 mm, amikacin R < 15 mm. The brakpoint for positive protease activity was set > 5mm (zero indicates growth but no transparency under the colony).

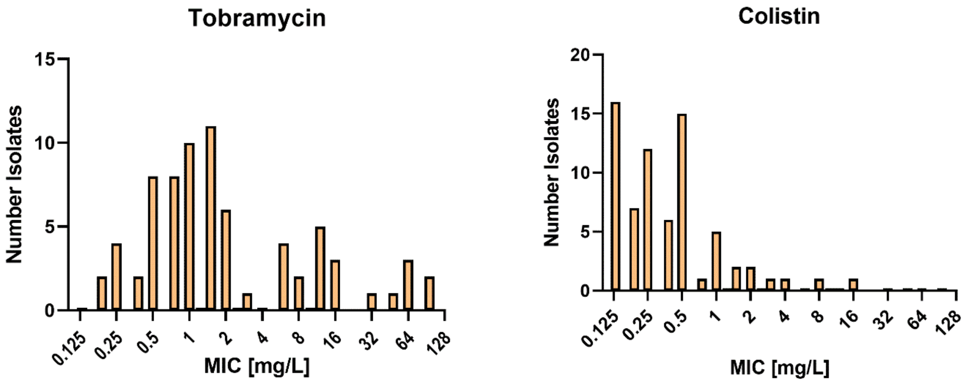

Figure S3. Distribution of the minimal inhibitory concentration (MIC in mg/L) of the CF-isolates assessed by antimicrobial susceptibility testing by E-test strips (Tobramycin) and broth microdilution (Colistin).

The clinical antimicrobial susceptibility breakpoints were applied according to the recent EUCAST guidelines (https://www.eucast.org/clinical_breakpoints) as followes: tobramycin R > 2 mg/L, colistin R > 4 mg/L.

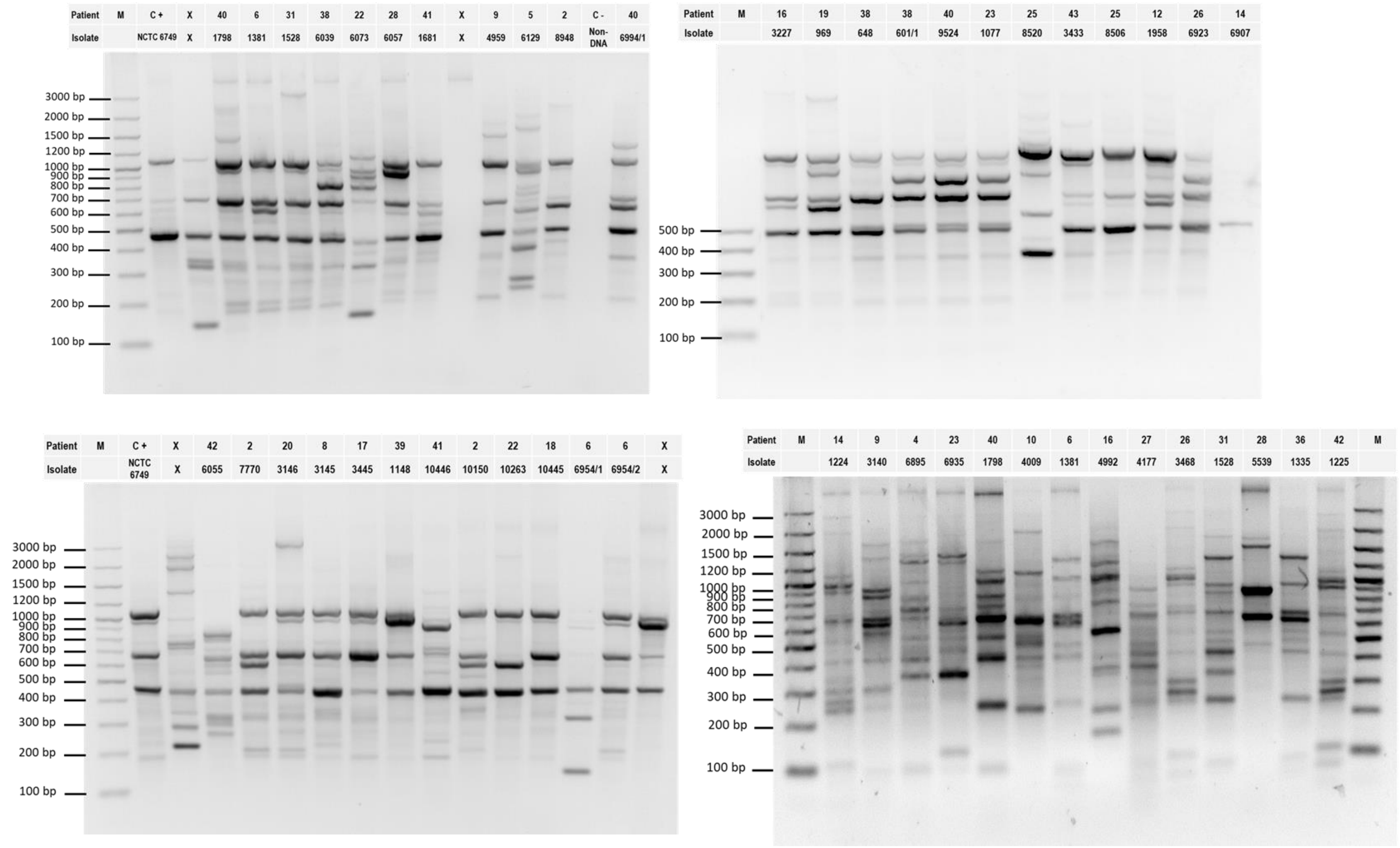

Figure S4. RAPD gels.

M = marker; C + = positive control *P. aeruginosa* NCTC 6749; C – = negative control, non-DNA or *E. coli* ATCC 25922; X = excluded from the analysis, replicate

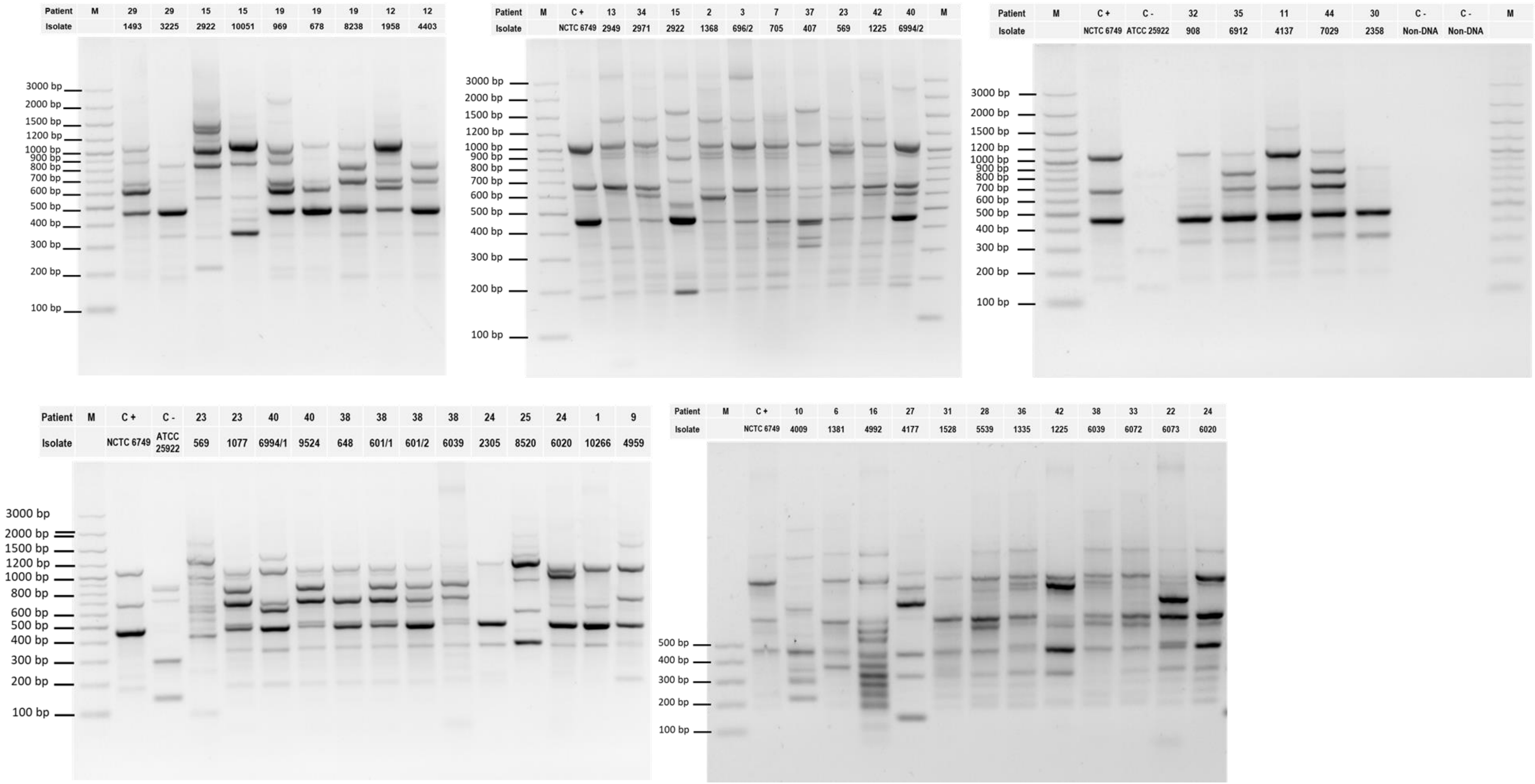

Figure S5. RAPD gels.

M = marker; C + = positive control P. aeruginosa NCTC 6749; C – = negative control, non-DNA or *E. coli* ATCC25922; X = excluded from the analysis, replicate

Table S1. Correlation analysis of the clinical parameters of the CF-cohort (n = 44) at first sampling expressed as Spearman's rank coefficients (confidence interval 95%).

	Age	Sex	BMI	AD	YT	HF	FEV_1	FVC	FEV_1/ FVC	QLT	Diabete s	PI	VF
Sex	0.04												
BMI	0.32	-0.19											
AD	0.27	0.20	0.26										
YT	0.84	0.09	0.14	0.12									
HF	-0.09	0.30	-0.22	0.01	0.02								
FEV_1	-0.05	0.12	0.51	0.40	-0.09	-0.46							
FVC	0.02	0.00	0.51	0.51	-0.06	-0.42	0.88						
FEV_1/FVC	-0.05	0.21	0.38	0.26	-0.03	-0.35	0.87	0.56					
QLT	-0.18	0.18	-0.42	-0.06	-0.09	0.43	-0.48	-0.36	-0.47				
Diabetes	0.31	-0.21	0.08	-0.04	0.16	0.18	-0.41	-0.38	-0.36	-0.05			
PI	-0.17	-0.09	-0.17	-0.45	-0.12	0.25	-0.23	-0.38	-0.04	0.13	0.33		
VF	-0.84	-0.09	-0.14	-0.12	-1.00	-0.02	0.09	0.06	0.03	0.09	-0.16	0.12	
F508del	0.02	-0.24	0.35	-0.07	0.07	0.01	0.06	0.03	0.11	-0.07	0.15	0.39	-0.07

BMI = body mass index, AD = age at diagnosis, YT = years under treatment, HF = hospitalizations frequency, FEV_1 = forced expiratory volume in 1 second, FVC = forced vital capacity, QLT = qualified for lung transplantation, PI = pancreatic insufficiency, VF = visit frequency, scale of P-values: dark grey ≤ 0.001 ≥ grey ≤ 0.01 ≥ light grey ≤ 0.05. The correlation significance (two-tailed) was assumed at level ≤ 0.05 (underlined in colour). Following assumptions were applied for the correlation: weak $r_s > 0.3$, moderate $r_s > 0.5$, strong $r_s > 0.7$, very strong $r_s > 0.9$.

Table S2. Correlation analysis of the *Pseudomonas aeruginosa* properties (n = 74) at first sampling from the CF-patient cohort expresses as Spearman's rank coefficients (r_s, confidence interval 95%).

	RAPD group	Biofilm ability	Mucoid	Number of resistances	4MRGN	PIP/TAZ	CAZ	MEM	CIP	CL	TB	AK	Prote-olysis	Swimming
**Biofilm ability**	0.32													
**Mucoidity**	0.20	-0.16												
**Number of resistances**	-0.16	0.03	-0.30											
**4MRGN**	0.01	0.02	-0.13	0.66										
**PIP/TAZ**	-0.09	-0.05	-0.21	0.69	0.67									
**CAZ**	-0.20	-0.08	-0.19	0.81	0.70	0.69								
**MEM**	-0.15	-0.08	-0.13	0.74	0.74	0.58	0.67							
**CIP**	-0.03	0.12	-0.17	0.74	0.56	0.35	0.52	0.58						
**CL**	-0.19	0.07	-0.17	0.35	0.03	-0.03	0.22	0.22	0.30					
**TB**	-0.11	0.06	-0.31	0.74	0.64	0.40	0.62	0.66	0.59	0.41				
**AK**	-0.18	0.08	-0.28	0.77	0.49	0.42	0.59	0.61	0.51	0.29	0.52			
**Proteolysis**	-0.11	-0.07	-0.07	0.01	0.03	0.10	0.02	0.06	0.04	-0.18	0.05	0.00		
**Swimming**	-0.04	-0.13	0.07	-0.20	-0.03	-0.05	-0.20	-0.11	-0.02	-0.35	-0.23	-0.19	0.27	
**Swarming**	-0.12	0.08	-0.15	-0.09	0.02	-0.04	-0.05	0.02	-0.01	-0.18	0.04	-0.10	0.40	0.41

4MRGN = resistant against tazobactam/piperacillin, ceftazidime, meropenem and ciprofloxacin, PIP/TAZ = piperacillin/tazobactam, CAZ = ceftazidime, MEM = meropenem, CIP = ciprofloxacin, CL = colistin, TB = tobramycin, AK = amikacin, scale of P-values: dark grey ≤ 0.001 ≥ grey ≤ 0.01 ≥ light grey ≤ 0.05. The correlation significance (two-tailed) was assumed at level ≤ 0.05 (underlined in colour). Following assumptions were applied for the correlation: weak $r_s > 0.3$, moderate $r_s > 0.5$, strong $r_s > 0.7$, very strong $r_s > 0.9$.

Patient No	Year of sampling	RAPD	Biofilm	Mucoid	Protease	Swimming	Swarming	PIP/TAZ	CAZ	MEM	CIP	COL	TB	AK
2	2014	1	3	0	1	1	1	0	0	0	0	0	0	0
	2015	2	0	1	0	1	1	0	0	0	0	0	0	0
	2015	1	0	1	0	1	1	0	0	0	0	0	0	0
	2016	1	0	0	0	0	0	0	0	0	0	0	1	0
6	2016	2	0	0	1	1	1	0	0	0	0	0	0	0
	2016	1	0	0	0	1	1	0	0	0	0	0	0	0
	2016	2	2	1	1	0	1	0	0	0	0	0	0	0
9	2016	0	1	1	1	1	1	0	0	0	0	0	0	0
	2016	2	2	0	1	1	1	1	0	1	1	0	1	0
12	2015	2	0	0	0	1	1	0	0	0	1	0	0	0
	2017	2	3	0	0	1	1	1	1	1	1	0	0	1
13	2016	1	0	1	1	1	0	1	1	1	0	0	0	2
	2016	0	0	0	0	0	0	1	1	1	0	0	0	1
14	2016	1	0	0	1	1	1	1	0	0	0	0	0	0
	2018	0	1	1	0	1	1	0	0	0	0	0	0	0
15	2016	0	2	0	0	0	0	0	0	1	1	1	1	1
	2017	0	0	0	1	1	1	0	1	0	1	0	0	1
16	2016	0	3	0	0	0	0	0	1	0	1	1	1	1
	2018	2	3	0	0	0	1	0	1	0	1		1	1
19	2014	2	1	0	0	0	0	0	0	0	0	0	0	0
	2014	2	1	0	0	1	1	0	0	0	0	0	0	1
	2018	2	3	0	0	0	0	0	0	0	0	0	0	1
22	2015	2	0	1	1	1	1	0	0	0	0	0	0	0
	2016	1	0	1	1	0	0	0	0	0	0	0	0	0
23	2016	1	1	0	1	0	1	1	1	1	0	0	1	1
	2016	0	0	0	1	0	1	0	0	2	1	0	0	2
	2017	2	3	0	1	0	1	0	0	0	0	0	0	0
24	2014	0	0	0	1	1	1	1	1	1	1	0	1	1
	2014		3	0	1	1	1	1	1	1	1	0	1	1
	2016	1	1	0	0	1	1	1	1	1	1	0	1	1
26	2016	0	0	0	1	1	0	1	1	1	2	0	1	1
	2018	2	2	0	1	0	0	1	1	1	1	0	1	1
28	2016	1	1	0	1	1	1	0	0	0	0	0	0	0
	2016	2	0	1	1	1	1	0	0	0	0	0	0	0
29	2016	2	0	0	0	1	0	0	0	0	0	0	0	0
	2018	2	2	0	0	0	0	0	0	0	1	0	0	0
33	2016	1	0	1	1	1	1	0	0	0	0	0	0	0
	2018	0	1	1	1	1	1	0	0	0	1	0	0	0
38	2013	2	0	1	1	1	0	0	0	0	0	0	0	1
	2014	2	3	0	1	1	1	0	0	0	0	0	0	0
	2014	2	2	1	1	1	1	0	0	0	0	0	0	0
	2016	2	0	1	0	0	0	0	0	0	0	0	0	0
40	2016	1	0	0	1	1	1	0	1	1	1	0	1	1
	2016	2	1	1	0	0	0	0	0	0	0	0	0	0
	2016	1	0	0	1	1	0	0	0	0	0	0	0	1
	2017	2	2	0	0	0	0	1	1	1	1	1	1	1
41	2015	1	0	0	1	1	1	0	0	0	1	0	0	0
	2016	2	3	0	1	1	1	0	0	0	0	0	0	0
42	2016	0	0	0	1	1	1	1	1	0	0	0	0	0
	2016	1	0	0	1	1	1	1	0	0	0	0	0	0

Figure S6. Visualisation of the phenotypic characteristics of the *P. aeruginosa* isolates in 20 patients with multiple samples.

The vertical black lines indicate isolates selected for further analysis from the same sample due to morphological differences. Colours indicate the coding as follows. RAPD: 0 = no specific cluster, 1 = cluster I, 2 = cluster II, no colour/code = no RAPD pattern assessed; biofilm: 0 = no biofilm formed, 1 = weak biofilm former, 2 = moderate biofilm former, 3 = strong biofilm former; swimming and swarming: 0 = negative, 1 = positive; Antibiotics: 0 = sensitive, 1 = resistant, 2 = intermediate; PIP/TAZ = piperacillin/tazobactam, CAZ = ceftazidime, MEM = meropenem, CIP = ciprofloxacin, COL = colistin, TB = tobramycin, AK = amikacin.
